# Clearing the chromatin roadblock: a dual strategy for H2A.Z depletion under high temperature

**DOI:** 10.1093/plcell/koag093

**Published:** 2026-03-26

**Authors:** Yu-Hung Hung

**Affiliations:** Assistant Features Editor, The Plant Cell, American Society of Plant Biologists; Spearhead Bio Inc, St. Louis, MO 63132, United States

The global average temperature has risen significantly, with the last decade being the warmest on record (NOAA 2025). The climate crisis severely impacts plant growth. While we can hide in the shade, turn on the air conditioning, or jump into a pool when temperatures soar, how do plants and crops survive—and even thrive—on a planet that is undeniably getting warmer?

Through a process termed thermomorphogenesis, plants can rapidly alter their growth patterns to cope with rising temperatures (Quint et al. 2023). When under high temperature, plants change their architecture by stretching the stem to push leaves away from the hot ground, catch cooler air, and increase airflow.

This rapid stretching requires the massive activation of specific genes, such as those in the auxin pathway, like *YUCCA8* (*YUC8*). To switch these genes on, the plant must clear molecular roadblocks from its DNA. Usually, activating a gene involves adding permissive histone acetylation tags to loosen the DNA. However, in a new study, **Xiaoyi Li and colleagues (Li et al. 2026)** identified that this pathway is dependent on HISTONE DEACETYLASE 9 (HDA9), an enzyme usually associated with the opposing activity (i.e. chromatin tightening).

Conventionally, histone deacetylases are categorized as transcriptional repressors that silence gene expression by removing activating histone marks (Liu et al. 2014). The involvement of HDA9 in heat-induced growth thus presents a biological paradox: how does a known silencer facilitate massive gene activation? Li et al. (2026) resolved this paradox by characterizing a multi-component protein complex involving the transcription factor PHYTOCHROME INTERACTING FACTOR 4 (PIF4), the histone variant H2A.Z, and the complex formed by FVE and HDA9.

When the temperature spikes, PIF4 acts as the master switch, binding to the promoters of target genes like *YUC8* (Koini et al. 2009). However, PIF4 cannot by itself access the DNA due to the presence of H2A.Z, a repressive histone H2A variant that tightly packages DNA into a closed chromatin state (Li et al. 2026). To overcome this, PIF4 recruits the FVE-HDA9 complex to specific genomic loci.

Analysis of *fve-4* loss-of-function mutants demonstrates that these plants are unable to activate relevant growth genes and initiate the temperature-dependent stretching response (Li et al. 2026). Although the mechanisms by which H2A.Z represses transcription remain incompletely understood, one potential mechanism is that it might act like a specialized spool, packing DNA much tighter than standard histones and locking genes down in a “tightly sealed vault.” For the plant to grow, H2A.Z must be evicted fast. In this new study, researchers demonstrated this by crossing the *fve* mutant with a plant lacking H2A.Z. This resulted in a complete genetic rescue; the plant grew normally under heat stress. This confirmed the essential role of FVE is controlling H2A.Z levels on chromatin. Mechanistically, HDA9 promotes activation indirectly by targeting the repressive structure itself. By removing specific acetylation marks that typically help the chromatin remodeling complex deposit H2A.Z, HDA9 prevents the installation of new repressive roadblocks (Li et al. 2026). This creates a chromatin environment hostile to gene silencing.

Moreover, the researchers showed that, in addition to inhibiting H2A.Z deposition, the INO80 chromatin remodeling complex actively removed previously deposited H2A.Z. INO80 acts as the “commanding general”; it not only performs active eviction of H2A.Z, but it also stabilizes the FVE-HDA9 complex to ensure that no new repressive material is being brought in during the heat stress period (Li et al. 2026).

In summary, Li and colleagues described a beautifully choreographed dual mechanism in response to high temperature (Fig. 1): inhibition of H2A.Z deposition and active eviction of H2A.Z. At ambient temperatures, the plant uses H2A.Z to keep growth genes repressed. At high temperatures, PIF4 recruits the FVE-HDA9 and INO80 complexes to clear the way for swift gene activation. This is the first plausible molecular explanation in plants for how a classic silencer like HDA9 is repurposed to promote stress-induced gene activation.

**Figure 1:**
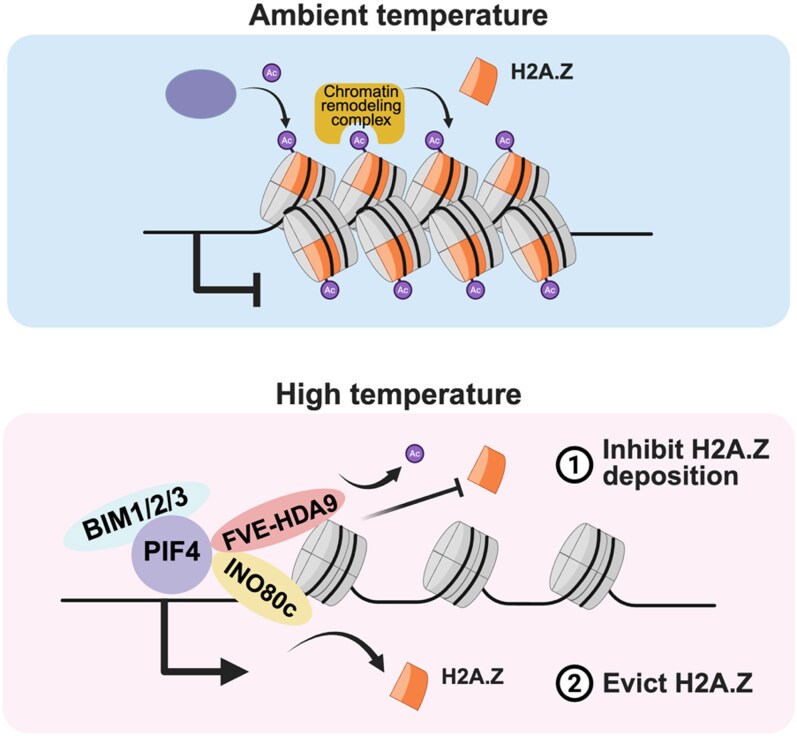
Model of dual mechanisms in response to high temperature. At ambient temperatures, the plant uses H2A.Z to keep growth genes repressed. At high temperatures, PIF4 recruits the FVE-HDA9 to inhibit H2A.Z deposition. PIF4 also recruits INO80 complexes to evict the previously deposited H2A.Z. The dual mechanism in response to heat clear the way for swift gene activation. Modified from Li et al. (2026), Figure 9. Created in https://BioRender.com. Figure credit: Yu-Hung Hung.

## Recent related articles in *The Plant Cell*:

- Verma et al. (2024) identified a regulatory mechanism in *Arabidopsis* where elevated temperatures activate the kinase MPK4, which subsequently phosphorylates the transcription factor PIF4 to repress ARP6 expression, leading to the eviction of the histone variant H2A.Z from target genes to promote hypocotyl elongation and early flowering.
- Kim et al. (2026) identified that the Polycomb-group protein VERNALIZATION INSENSITIVE 3-LIKE 1 (VIL1) promotes growth retardation in Arabidopsis at low ambient temperatures by suppressing the expression of the cold-response regulator CBF1 through dynamic chromatin remodeling and H3K27me3 deposition at the CBF gene cluster.

## Data Availability

No new data were generated or analysed in support of this research.

## Dive Curated Terms

The following phenotypic, genotypic, and functional terms are of significance to the work described in this paper:

CBF1 Gramene: AT4G25490
CBF1 Araport: AT4G25490

ARP6 Gramene: AT3G33520
ARP6 Araport: AT3G33520

MPK4 Gramene: AT4G01370
MPK4 Araport: AT4G01370

HDA9 Gramene: AT3G44680
HDA9 Araport: AT3G44680

INO80 Gramene: AT3G57300
INO80 Araport: AT3G57300

FVE Gramene: AT2G19520
FVE Araport: AT2G19520

VIL1 Gramene: AT3G24440
VIL1 Araport: AT3G24440
